# 固相萃取吸附材料在植物激素样品前处理中的应用新进展

**DOI:** 10.3724/SP.J.1123.2021.03045

**Published:** 2021-12-08

**Authors:** Shuting LIN, Qingqing DING, Wenmin ZHANG, Lan ZHANG, Qiaomei LU

**Affiliations:** 1.福州大学福建省高校测试中心, 福建 福州 350116; 1. Fujian College Association Instrumental Analysis Center of Fuzhou University, Fuzhou 350116, China; 2.福州大学环境与资源学院, 福建 福州 350116; 2. College of Environment and Resources, Fuzhou University, Fuzhou 350116, China; 3.食品安全与生物分析教育部重点实验室, 福州大学化学学院, 福建 福州 350116; 3. Key Laboratory for Analytical Science of Food Safety and Biology (Ministry of Education), College of Chemistry, Fuzhou University, Fuzhou 350116, China; 4.闽江师范高等专科学校化学与生物工程系, 福建 福州 350108; 4. Division of Chemical and Biological Engineering, Minjiang Teachers College, Fuzhou 350108, China

**Keywords:** 吸附材料, 植物激素, 样品前处理, 固相萃取, 综述, adsorption material, plant hormone, sample pretreatment, solid phase extraction (SPE), review

## Abstract

植物激素在植物生长过程中具有重要作用,调节植物生长、发育及抗逆的各个过程。植物激素超微精准定量分析一直是植物生理学研究的瓶颈问题。植物激素的准确、高效检测目前大多是基于液相色谱-串联质谱联用技术。样品前处理是植物激素色谱-质谱分析中必不可少的一个步骤,直接影响后续检测方法的灵敏度和准确性。在植物激素各种前处理方法中,固相萃取(SPE)技术应用非常广泛。在萃取小柱基础上发展了多种新形式(分散固相萃取、磁性固相萃取、固相微萃取等,称之为SPE相关方法)。在上述SPE相关方法中,吸附材料的选择均是关键因素,决定了样品前处理过程的目标物提取、净化和富集效果。碳基材料(包括碳纳米管、石墨烯、碳氮化合物等)和有机骨架材料(包括金属有机骨架、共价有机材料)拥有结构可设计、比表面积大、稳定性良好等特性,非常适合作为吸附材料。分子印迹聚合物和超分子化合物依靠主-客体特异性分子识别作用,能显著提高样品前处理方法的选择性。本文重点针对植物激素样品前处理中的SPE技术,综述了近5年来上述几类功能化吸附材料的最新应用进展,并对其发展趋势进行展望。

## Abstract (en)

Plant hormones (PHs) are of significance in plant growth, as they regulate the various processes related to plant growth, development, and resistance. Sensitive and precise quantitative analysis of PHs is a bottleneck in plant science research. Currently, liquid chromatography-tandem mass spectrometry is used for the accurate and efficient detection of PHs. Sample pretreatment is an indispensable step in the chromatography-mass spectrometry analysis of PHs because it directly affects the sensitivity and accuracy of subsequent detection methods. Among various pretreatment methods for PHs, solid phase extraction (SPE) is the most widely used. Various new types of SPE, such as dispersive SPE, magnetic SPE, and solid phase microextraction, have been developed by modifying the extraction cartridge. The choice of adsorption material is the key factor in the abovementioned SPE methods, which has a decisive effect on the extraction, purification, and enrichment effects of the target substance in the sample pretreatment process. Carbon-based materials, including carbon nanotubes, graphene, carbon and nitrogen compounds, as well as organic frameworks, including metal organic frameworks and covalent organic materials, are suitable adsorption materials because of their designable structure, large specific surface area, and good stability. Molecularly imprinted polymers and supramolecular compounds show specific molecular recognition based on host-guest interactions, which can significantly improve the selectivity of sample pretreatment methods. In this paper, SPE-related technology and the abovementioned types of functionalized adsorption materials in the pretreatment of PHs prevalent in the past five years have been reviewed. The related development trends are also summarized.

植物激素(plant hormones, PHs)在植物生长过程中发挥着重要作用,调节植物生长、发育及抗逆的各个过程。按照生理功能以及化学结构不同,PHs可分为生长素类(auxin, Aux)、赤霉素类(gibberellins, GAs)、脱落酸(abscisic acid, ABA)、细胞分裂素类(cytokinins, CTKs)、乙烯(ethylene, ETH)、油菜素内酯类(brassinosteroids, BRs)、独脚金内酯类(strigolactones, SLs)和茉莉酸类(jasmonic acids, JAs)等(基本信息见表1)^[1,2]^。据报道,Aux、CTKs、ABA、JAs等含量约1~100 ng/g FW(鲜重)之间,GAs低于0.1 ng/g FW, BRs含量最低(少于1 pg/g FW)^[3]^。同时,各类PHs又因为结构和官能团差异,显示不同的酸碱性。例如,酸性激素包括Aux、GAs、ABA、JAs、SA等几大类,ETH、BRs和SLs总体表现为中性,CTKs由于带有数目较多的氨基而表现为弱碱性。

**表1:** 植物激素基本信息表

Type of plant hormones	Introduction	Representative structure	Common compounds and their abbreviations
Auxin (Aux)	Discovered in 1880, promote growth at low concentration while inhibit growth at high concentration.	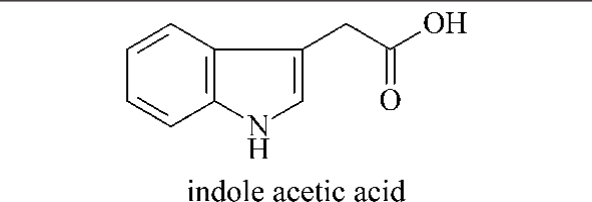	indole acetic acid (IAA) 4-chloroindole acetic acid (4-Cl-IAA) indole butyric acid (IBA) indole propionic acid (IPA) naphthalene acetic acid (NAA) 2-naphthoxyacetic acid (2-NOA) 2,4-dichlorophenoxyacetic acid (2,4-D)
Gibberellins (GAs)	Discovered in 1934, promote cell elongation and germination, relieve seed dormancy, etc.	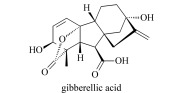	gibberellic acid (GA_3_) gibberellin 1 (GA_1_) gibberellin 4 (GA_4_) gibberellin 7 (GA_7_)
Cytokinins (CTKs)	Discovered in 1955, promote cell division and bud differentiation, delay senescence, etc.	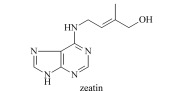	zeatin (ZT) dihydrozeatin (DHZ) isopentenyl adenine (iP) zeatin riboside (ZR) kinetin (KT) 6-benzylaminopurine (BA)
Abscisic acid (ABA)	Discovered in 1963, promote dormancy, close stomata, increase stress resistance, etc.	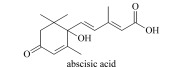	abscisic acid
Ethylene (ETH)	Discovered in 1901, the only gaseous plant hormone, promote organ shedding, stomata closure and fruit ripening, etc.	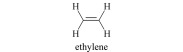	ethylene
Brassinolides (BRs)	Discovered in 1979, promote cell elongation and division, enhance stress resistance, etc.	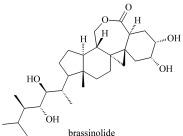	brassinolide (BL) 2,4-epibrassinolide (2,4-epiBL)
Strigolactones (SLs)	Discovered in 1966, promote the symbiosis of plants and soil microorganisms, etc.	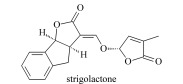	strigolactone (SL) 5-deoxystrigol
Jasmonic acids (JAs)	Discovered in 1962, close stomata and enhance stress resistance.	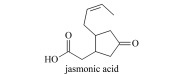	jasmonic acid (JA) methyl jasmonate (MeJA)
Salicylic acid (SA)	Discovered in 1992, enhance stress resistance.	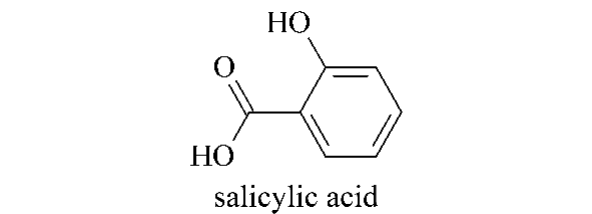	salicylic acid

鉴于植物内源激素含量少,迫切需要高效灵敏的分析手段来准确测定PHs浓度。液相色谱-质谱系列技术(包括LC-MS、LC-MS/MS、UPLC-MS/MS)具有分离快速、鉴定准确等优势,在有机物定性、定量检测中应用广泛,已成为目前PHs高灵敏度分析、交叉作用研究的最主流方法^[4,5,6]^。

## 1 植物激素的样品前处理方法

植物组织中PHs含量少且浓度范围跨度大,又含有糖类、蛋白、色素等杂质成分,分析前要选择适当的提取、纯化和富集步骤。根据“相似相溶原理”, 80%甲醇是PHs分析最常用的提取剂。用有机溶剂提取内源激素时,植物基质中其他组分会被一并提取,故需要进一步净化。PHs的纯化方法包括液液萃取、固相萃取和各种微萃取技术^[7,8]^。此外,PHs早期研究中,受到前处理方法、检测仪器灵敏度等因素限制,常用到几克甚至几十克植物材料,这对于某些珍贵样品不利,同时势必要消耗更多的吸附材料和有机溶剂。

固相萃取技术(solid phase extraction, SPE)是PHs分离的经典前处理方法^[9]^,具有溶剂消耗少、适用范围广等优势,但传统SPE小柱存在容易堵塞、操作耗时等不足。基于SPE类似原理,衍生发展了一些新技术,包括基质分散固相萃取(matrix solid phase dispersion extraction, MSPD)、分散固相萃取(dispersive solid phase extraction, dSPE)、磁性固相萃取(magnetic solid phase extraction, MSPE)、移液器尖端固相萃取法(pipette tip solid phase extraction, PT-SPE)、固相微萃取(solid phase microextraction, SPME)等改进形式。上述方法在PHs样品前处理中都有应用。吸附剂材料是影响SPE系列方法萃取效率和选择性的关键因素,也直接影响PHs检测的回收率和灵敏度等重要指标。

## 2 植物激素的固相萃取吸附材料

本文重点针对SPE及其有关技术,讨论碳基材料、有机骨架化合物以及选择性更强的分子印迹聚合物、超分子化合物等几类功能化吸附材料在PHs样品前处理中的最新应用。

### 2.1 碳基材料

碳元素存在许多同素异形体。碳纳米管、石墨烯等材料具有比表面积高、化学稳定性好、易于功能化修饰等性能,作为PHs的固相萃取吸附剂研究非常活跃。

2.1.1 碳纳米管

碳纳米管(carbon nanotubes, CNTs)是由石墨片层以*sp*^2^杂化围绕中心轴卷曲而成,有单壁碳纳米管(single-walled carbon nanotubes, SWCNTs)和多壁碳纳米管(multiwalled Carbon Nanotubes, MWCNTs)两类。自1991年发现以来,CNTs就以其良好的机械强度、吸附能力和化学稳定性等特点在样品前处理领域中应用广泛^[10]^。

CNTs对有机物有很强的亲和力,尤其对结构中含有苯环、萘环的目标物。Wang等^[11]^首次将MWCNTs用作SPE吸附剂来萃取豆芽样品中IBA和NAA。实验选择80 mg MWCNT吸附材料,1 mL甲醇-水溶液(90∶10, v/v)洗脱,结果表明,提取和净化效果明显优于商品化C_18_-SPE小柱。该HPLC方法回收率大于81.4%,检出限低至3.0 ng/mL。

固相微萃取(SPME)是一种非溶剂型微萃取技术。其中,中空纤维(hollow fiber, HF)膜保护萃取方式既能保证吸附材料免受复杂基质的干扰,又能利用HF孔洞结构提高目标物萃取的选择性。国内师彦平课题组利用CNTs具有表面积大、吸附能力高的结构特点,采用溶胶-凝胶技术和表面活性剂辅助分散法将CNTs固定在HF壁孔中,制备成一种新型的微萃取材料-碳纳米管增强的中空纤维(CNTs-HF),用于一系列化合物的SPME萃取富集。在PHs应用上,该课题组开发了基于*β*-环糊精修饰碳纳米管的中空纤维固相微萃取技术(CNTs-*β*-CD-HF-SPME)^[12]^。与单纯HF材料相比,CNTs-*β*-CD-HF材料孔洞结构明显减少,但该材料的富集效果和选择因子却非常理想,对NAA和2-NOA的富集倍数分别为275和283倍,远高于CNTs-HF材料(153和118倍)和常规HF材料(34倍和95倍),表明*β*-环糊精和CNTs的加入,增强了材料与目标物之间的疏水作用和尺寸效应。随后,课题组又相继提出碳纳米管增强的中空纤维电膜萃取(CNTs-HF-EME)^[13]^。在该研究中,以电迁移作为萃取动力来加快传质过程,加入CNTs有助于提高萃取性能和方法灵敏度,对番茄样品中NAA和NOA进行检测,样品提取时间缩短至30 s。在前期研究的基础上,又改进提出了N掺杂碳纳米管的中空纤维固相微萃取法(N-doped CNTs-HF-SPME)^[14]^。通过N掺杂引入了更多碱性位点和正电荷表面,对2种Aux的萃取效果顺序为N-doped CNTs-HF-SPME>CNTs-HF-SPME>HF-SPME,证实N掺杂碳纳米管能改善萃取性能。

乙烯是唯一的一类气态植物激素,具有调控果实成熟和叶片衰老的作用,也是一种被公认为可安全处理农产品的激素组分,在水果采摘前和收获后都有广泛应用。CNTs材料在ETH检测上有不少应用^[15,16,17]^。2020年,Chen等^[17]^制备了一种基于单壁碳纳米管的纳米复合材料(Cu-Tm-coated MoS_2_/SCNT),用于实时检测不同水果表面的ETH释放量,检测灵敏度低至0.1 ng/mL。

2.1.2 石墨烯

作为一种新型二维碳纳米材料,石墨烯(graphene, G)具有蜂窝状的晶格结构^[18]^。G及其复合材料在样品前处理中的应用与CNTs类似,比表面积大、*π-π*相互作用强等特性使其对有机物表现出优异吸附性。在吸附过程中直接使用G容易发生团聚,故在实际应用中,通过改性修饰来提高G材料的吸附选择性和分散性。氧化石墨烯(graphene oxide, GO)作为石墨烯的重要衍生物之一,其制备过程大致如下:以Hummers及其改进方法为例^[19]^,反应体系由石墨、NaNO_3_、KMnO_4_以及浓H_2_SO_4_组成,产物经冷冻干燥、超声处理后获得GO。GO表面含有亲水性含氧基团(例如羧基、羟基等),更容易被结构修饰。

Wang等^[20]^分别制备了SiO_2_、GO硅球(GO/SiO_2_)、三(吲哚基)甲烷复合GO硅球(Ntim-GO/SiO_2_)等3种材料作为SPE小柱的吸附剂,用于SA萃取。研究者发现Ntim-GO/SiO_2_与酸性目标物之间存在更多的氢键和疏水作用位点,同时Ntim与GO形成协同效应,因此萃取效果最高。将GO进一步还原,获得的还原氧化石墨烯(reduced graphene oxide, RGO)同样含有少量含氧官能团,有利于提高吸附作用位点和制备成各种复合材料。Li等^[21]^采用化学共沉淀法制备磁性G,再将*β*-CD修饰到其表面,合成Fe_3_O_4_/RGO@*β*-CD作为MSPE技术的吸附剂。基于*β*-CD的主-客体分子识别能力,对具有吲哚环的IAA和IPA、具有萘环的NAA和2-NOA表现出理想的特异性吸附,选择因子介于1.01~2.44之间。2018年,Chen等^[22,23]^合成类似材料,研究了蔬菜样品中多种PHs的残留情况。引入离子液体(ionic liquid, IL)可提供较多的氢键、疏水、静电作用位点,并显著提高萃取效果。Cao等^[24]^制备Fe_3_O_4_@SiO_2_/GO/*β*-CD/IL复合材料,比表面积和孔体积分别为55.44 m^2^/g和0.22 cm^3^/g,孔径平均为15.91 nm,萃取效果优于Fe_3_O_4_@SiO_2_/GO、Fe_3_O_4_@SiO_2_/GO/*β*-CD以及Fe_3_O_4_@SiO_2_/GO/IL。随着原位SPME发展,Fang等^[25]^制备了一种基于C_18_和二烯丙基二甲基氯化铵的氧化石墨烯(C_18_@GO@PDDA)复合材料,首次实时追踪了镉胁迫下芦荟样品中SA的浓度变化。

作为传统SPE小柱的微型化改进装置,移液器尖端固相萃取法(PT-SPE)需要的吸附剂和有机溶剂量更少,更符合绿色化学要求。Wang等^[26]^采用Hummers方法,以GO、聚吡咯和过硫酸钾等混合物合成了一种新型氧化石墨/聚吡咯(GO/Ppy)泡沫材料。加入聚吡咯能扩大材料的比表面积、增加亲水基团。3.0 mg材料吸附填充于50 μL移液器枪头中,经过2.0 mL甲醇-氨水(80∶20, v/v)洗脱后进行HPLC-DAD检测,3种Aux回收率大于89.4%。2018年,同课题组通过点击化学制备了新型离子液体-硫醇-氧化石墨烯(IL-TGO)^[27]^作为PT-SPE吸附剂,加入硫醇和离子液体可防止石墨烯片聚集。方法对IAA、NAA和2,4-D的吸附量分别为21.7、41.8和30.7 μg/mg,萃取机理涉及离子交换、静电、氢键和*π-π*堆积等多重作用。

dSPE技术于2003年首次提出^[28]^,将吸附剂直接分散在样品溶液中,具有萃取快速、样品消耗少等优点。Zhang等^[29]^将氧化石墨烯SiO_2_纳米复合材料(SiO_2_@GO)作为dSPE吸附剂,优化了吸附剂用量、样品溶液pH值、解吸溶液等参数,最终在拟南芥、桃子等5种植物样品中实现了4种Aux的快速提取纯化。

磺化石墨烯(sulfonated graphene, SG)也可以避免单纯G的团聚现象,有效增强材料的稳定性。Ling等^[30]^将聚(3,4-乙烯二氧噻吩)(PEDOT)和SG组成的导电复合膜(PEDOT-SG)电化学沉积在碳纤维束上,基于SPME方法来提取JA和MeJA,富集倍数超过420倍。

2.1.3 石墨炭黑材料

石墨炭黑(graphitized carbon black, GCB)是一类典型的疏水性材料,由炭黑在惰性气体中高温煅烧获得。基于GCB能与植物叶绿素存在极强的*π-π*作用,故在PHs样品前处理中常作为dSPE、QuEChERS等方法的净化吸附剂^[31,32]^,用于去除植物基质中其他干扰物。

冯钰锜团队^[33]^选择GCB作为dSPE方法的分散剂和吸附剂,建立了dSPE-UPLC-MS/MS高通量分析包括Aux、CTKs、GAs、JAs等在内的54种PHs,方法回收率为80.3%~120.4%,并成功地用于研究水稻中内源激素的时空分布。Sutcharitchan等^[34]^以39种PHs为研究对象,选择C_18_、伯仲胺(primary secondary amine, PSA)、GCB等材料中的一种或组合几种,作为dSPE吸附剂,发现C_18_对所有目标物的保留行为较弱,PSA对酸性和极性激素有较强的亲和力,而GCB对CTKs吸附明显,导致该类激素的洗脱回收率低于10%。2020年,Jiang等^[35]^选择聚乙烯基吡咯烷酮-石墨炭黑(PVPP-GCB)作为dSPE吸附剂,再结合阳离子交换SPE方法来净化茶叶基质,用于13种酸性PHs及其类似物的分离检测,探讨了生物(害虫侵害)和非生物(光照)胁迫下不同时期茶叶样品中激素含量变化,对深入了解PHs对茶叶生长发育及胁迫响应的机制具有指导意义。

2.1.4 碳氮材料

碳、氮两种元素通过*sp*^2^杂化形成的层状碳基材料称为氮化碳。石墨化氮化碳(graphitized carbon nitride, g-C_3_N_4_)兼具离域大*π*键、良好分散性和生物兼容性,是理想的吸附材料^[36]^。Qiangba等^[37]^合成g-C_3_N_4_@SiO_2_复合材料时发现,加入HCl对材料进行质子化可提高萃取效率,对IBA等目标物的吸附容量在500~558.8 μg/g范围,回收率达到81.1%~121.8%。Xie等^[38]^制备了基于Au掺杂g-C_3_N_4_材料(g-C_3_N_4_/Au)的分子印迹聚合物膜,用于特异性识别GA_3_,方法灵敏度高、选择性好。

2020年,本课题组^[39]^基于氧化碳氮材料(oxygenated carbon nitride, OCN),合成了一种鱼鳞状磁性纳米复合材料(Co@Co_3_O_4_/OCN)。通过在OCN纳米片上原位掺杂氮,增加材料的吸附位点。该材料表现出良好的分散性,用作MSPE高效吸附剂实现了3种Aux的吸附萃取。选择对镉有明显富集作用的紫苏植物作为实际样品,揭示了镉胁迫下不同部位Aux的含量响应规律。

2.1.5 其他碳材料

碳纤维(carbon fibers, CFs)材料具有良好的化学稳定性。2019年,Zou等^[40]^合成了新型碳纤维涂层(CCFs),将*N*,*O*-双(三甲基甲硅烷基)三氟乙酰胺试剂直接在纤维上衍生,采用GC-MS技术分析小麦样品中JA、IAA、ABA。该铅笔型CCFs-SPME装置可实现提取/衍生化、进样等过程快速完成。次年,同课题组开发了碳纤维离子液体材料(CFs-IL)作为SPME涂层^[41]^,结合LC-MS/MS法高灵敏萃取和检测13种激素(包括中性、酸性和碱性组分),检出限为1.3~55.7 pg/mL。

过渡金属碳化物材料具有类G结构,统称为MXene,研究正兴起。Ti_3_C_2_ MXene具有二维微裂纹结构,比表面积大、孔隙率高,适合用于PHs提取。2021年,Luo等^[42]^制备了磁性吸附剂Fe_3_O_4_@Ti_3_C_2_@*β*-CD,结合原位衍生和UPLC-MS/MS技术,在单一油菜籽(4~6 mg)中获得了12种PHs超微定量和时空分布的信息。碳基材料在PHs固相萃取系列方法中的应用见表2。

**表2:** 碳基材料在植物激素固相萃取系列方法中的应用

Adsorbent	Analytical method	Analyte	Detection limit		Sample	Reference
MWCNTs	SPE-HPLC	IBA, NAA	1.2-3.0	ng/mL	bean sprouts	[11]
CNTs-β-CD-HF	SPME-HPLC	NAA, 2-NOA	0.8-1.5	ng/g	tomato	[12]
CNTs-HF	EME-HPLC	NAA, 2-NOA	1.5-2.0	ng/g	tomato	[13]
N-doped CNTs-HF	SPME-HPLC	NAA, 2-NOA	1.0-1.5	ng/g	tomato	[14]
Ntim-GO/SiO_2_	SPE-HPLC	SA	0.50	ng/mL	honey	[20]
Fe_3_O_4_/RGO@β-CD	MSPE-HPLC	NAA, 2-NOA	0.67	ng/g	tomato	[21]
Fe_3_O_4_@SiO_2_/GO/ β-CD	MSPE-LC-MS/MS	NAA, 2,4-D, etc	0.04-0.28	ng/g	cucumber, tomato, sprouts, asparagus lettuce and cabbage	[23]
Fe_3_O_4_@SiO_2_/GO/ β-CD/IL	MSPE-LC-MS/MS	Aux, CTKs	0.01-0.18	ng/g	cabbage, cucumber, tomato, eggplant and sprouts	[24]
C_18_@GO@PDDA	SPME-HPLC	SA, 3-SA	1.8-2.8	ng/mL	aloe	[25]
GO/Ppy	PT-SPE-HPLC	IPA, IBA, NAA	1.2-1.7	ng/g	papaya	[26]
IL-TGO	PT-SPE-HPLC	IAA, NAA, 2,4-D	4.0-26	ng/g	bean sprouts	[27]
SiO_2_@GO	dSPE-HPLC	Aux, ABA	30-50	ng/mL	arabidopsis, peach, cucumber, ginger and tomato	[29]
PEDOT-SG	SPME-HPLC	JA, MeJA	0.05-0.5	ng/mL	winter flower	[30]
GCB	dSPE-LC-MS/MS	Aux, CTKs, GAs, JAs, SA	0.02-31.09	fmol	rice	[33]
C_18_, PSA, GCB	dSPE-LC-MS/MS	ABA, Aux, GAs, CTKs	<10	ng/g	Chinese medicine	[34]
PVPP-GCB	dSPE-LC-MS/MS	ABA, Aux, GAs	0.1-120.1	ng/g	tea	[35]
g-C_3_N_4_@SiO_2_	SPE-HPLC	Aux, SA	1.9-5.7	ng/mL	coconut water	[37]
Co@Co_3_O_4_/OCN	MSPE-LC-MS/MS	IAA, IPA, IBA	0.2-4.0	pg/mL	perilla frutescens	[39]
CCFs	SPME-GC-MS	JA, IAA, ABA	0.04-0.17	ng/mL	tomato	[40]
CFs-IL	SPME-LC-MS/MS	Aux, ABA, GAs, CTKs, SA, JAs, BRs	1.3-55.7	pg/mL	tomato	[41]
Fe_3_O_4_@Ti_3_C_2_@β-CD	MSPE-UPLC-MS/MS	GAs, Aux, ABA, JA	2.18-45.39	pg/mL	oilseed	[42]

### 2.2 金属有机框架材料

金属有机框架材料(metal organic frameworks, MOFs)是一类由金属离子及有机配体自组装而成的多孔材料,分为IRMOFs系列、ZIFs系列、UiO系列以及MILs系列^[43]^。这种无机-有机杂化材料具有结构多样化、比表面积大、表面易修饰、孔径尺寸可调节等特点,适合作为PHs样品前处理的固体吸附剂。

2.2.1 IRMOFs系列

IRMOFs系列材料具有立方体结构、孔容积较大,其中MOF-5(也称IRMOF-1)最具有代表性。Hu等^[44]^采用共价键和法首次制备磁性MOF-5材料,对非极性(多环芳烃)和极性化合物(赤霉素类)均显示出优异的富集能力。他们先对Fe_3_O_4_微球进行羟基化和氨基化改性,制备成Fe_3_O_4_-NH_2_磁球;然后采用六水合硝酸锌以及对苯二甲酸合成MOF-5材料;紧接着,将上述MOF-5和Fe_3_O_4_-NH_2_在120 ℃条件下水热反应10 h,即制得磁性MOF-5材料。结合LC-MS/MS方法,实现了植物样品中GA_3_、GA_1_、GA_7_和GA_4_的高灵敏分析。

Yaghi等^[45]^合成了手性MOF-520材料,用于醇类、苯酚类和羧酸类等16种代表性化合物(包括JA和GA_3_两类激素异构体)的分析。该方法精确计算了2种GA_3_异构体中部分碳-碳单键、双键键角的细微差别,还实现了(-)-JA和(+)-JA异构体的选择性区分,改变了JA绝对构型及晶体结构研究相对缺乏的现状。

2.2.2 ZIFs系列

ZIFs系列是一类沸石咪唑酸酯骨架的MOF,经典代表性物质ZIF-8具有良好的化学稳定性、极大的比表面积和适宜的小孔尺寸,非常适合吸附小分子化合物。You等^[46]^通过一锅煮法制备了沸石咪唑酯骨架-8/聚(甲基丙烯酸甲酯-乙二醇二甲基丙烯酸酯) (ZIF-8/poly(MMA-EGDMA))整体包覆的搅拌棒吸附萃取(SBSE)涂层,用于分析苹果和梨中的5种PHs,检出限为0.11 ng/mL。与商品化聚乙二醇(PEG)和聚(MMA-EGDMA)涂层相比,该涂层具有氢键、疏水和静电等多重相互作用,提取效率更高。2018年,Xu等^[47]^通过微波辅助加热法制备了ZIF-8@SiO_2_核-壳微球,发现经3次涂覆后,材料的比表面积高达977.058 m^2^/g,用于Aux和CTKs两类激素的萃取,回收率介于82.7%~111.0%。

师彦平团队合成了基于聚沸石咪唑盐-67(ZIF-67)骨架的磁性羟基化多壁碳纳米管复合材料(Fe_3_O_4_-MWCNTs-OH@poly-ZIF67),对多苯环和多羧基结构的分析物表现出良好的萃取能力和选择性^[48]^,成功测定了水果样品中痕量NAA残留。在ZIF-67研究基础上,该课题组通过室温化学沉淀法首次制备了圆柱形铕沸石咪唑骨架(ZIF-Eu)材料,并将羧基多壁碳纳米管(MWCNTs-COOH)缠绕在ZIF-Eu周围,由此获得磁性纳米复合材料Fe_3_O_4_-MWCNTs-COOH/ZIF-Eu。该材料对NAA萃取效果理想,对苯、酚和酮类化合物萃取能力较差。经对比发现,该复合材料的吸附性能优于单纯Fe_3_O_4_-MWCNTs-COOH和ZIF-Eu两种材料^[49]^。2021年,He等^[50]^将ZIF-67煅烧生成磁性Co和N掺杂的多孔碳结构(Co@NC),该前体既能提供石墨碳,还可作为微孔有机网络材料(MON-2NH_2_)核-壳结构的核心。最终制备钴掺杂的碳氮-微孔有机网络(Co@NC-MON-2NH_2_)复合材料作为MSPE吸附剂,用于蔬菜中SA、NAA等4种激素的分析,证实MON是一种很有前景的新型吸附材料。

2.2.3 UiO系列

UiO系列由金属锆与有机配体配位形成,与ZIF系列相比,中心离子与配体之间的结合力更强、稳定性更好。Liu等^[51]^通过溶剂热法合成UiO-67,首次作为dSPE吸附剂,同时富集水果样品中的8种PHs。2018年,本课题组在MOFs材料UiO-66基础上,合成了聚丙烯腈静电纺丝(UiO-66/PAN)纳米纤维^[52]^,纤维的三维网络结构有利于萃取过程的快速传质。结合PT-SPE-HPLC法用于4种Aux富集和检测,方法有机溶剂用量少,检出限介于0.01~0.02 ng/mL。

2.2.4 MILs系列

MILs系列MOFs是一类三维的、具有类似沸石拓扑结构的多孔金属-羧酸化合物。2020年,Qin等^[53]^制备了介孔二氧化硅纳米颗粒-金属有机骨架(MSN@MIL-101(Fe))复合材料,结合dSPE方法萃取5种PHs,最终在绿豆芽中检出IAA和ABA。方法灵敏度和富集效果都明显优于上述SiO_2_@GO^[29]^以及UiO-67材料^[51]^。MOFs材料另一个很重要的应用领域是气体吸附与分离,在气态ETH萃取方面有初步尝试。Zhang等^[54]^合成了一种开放金属位点的复合材料(MIL-101-Cr-SO_3_Ag),发现金属位点和*π*络合物的引入能提高ETH吸附的选择性。

2.2.5 其他MOFs材料

MOF-199(也称HKUST-1)材料普遍基于1,3,5-均苯三甲酸和硝酸铜配位制备,具有不饱和金属位点和优良稳定性。Zhang等^[55]^制备了新型杂化涂层MOF-199/CNTs用于几种水果样品中痕量ETH无损分析。实验结果表明,掺杂CNTs可提高材料的耐腐蚀性和富集能力,材料萃取容量高达80 μg/L,检出限为0.016 μg/L。

2016年,Zhang等^[56]^合成了基于对苯二甲酸铜的金属骨架(CuTPA MOF)复合材料(表面积为708 m^2^/g,总孔体积为0.39 cm^3^/g)。在4 L容器中,每50 mg MOF材料可吸收和释放ETH高达654 μL/L。该研究为水果采摘后应用ETH诱导和促进果实成熟提供支持。随后,该课题组开发了另一种基于铝盐修饰金属骨架材料的新型纳米颗粒(Al-MOF)^[57]^,用于ETH的存储和释放研究。该材料在低于101.3 kPa的不同压力下,对ETH具有恒定的吸附速率。在标准大气条件下,ETH吸收率为41.0 cm^3^/g,与MIL-101材料的吸附能力(42.0 cm^3^/g)相当。这项技术能实现ETH存储和释放的精确控制,有望推广应用于农业和食品加工行业。MOFs材料用于SPE系列方法的汇总见表3。

**表3:** 金属有机骨架材料在植物激素固相萃取系列方法中的应用

Adsorbent	Pretreatment	Analyte	Recovery/%		Sample	Reference
MOF-5	MSPE	GAs	71.8-	127.4	buckwheat seedling	[44]
ZIF-8/poly(MMA-EGDMA)	SBSE	ABA, Aux, SA	82.7-	111.0	apple, pear	[46]
ZIF-8@SiO_2_	dSPE	Aux, CTKs	73.2-	89.6	navel orange	[47]
Fe_3_O_4_-MWCNTs-OH@poly-ZIF67	MSPE	NAA	92.4-	96.3	apple	[48]
Co@NC-MON-2NH_2_	MSPE	SA, NAA, 1-NOA	77.9-	114.4	tomato, mung bean sprout, cucumber	[50]
UiO-67	dSPE	GA_3_, Aux	89.3-	102.3	grapefruit, apple, pear	[51]
UiO-66/PAN	PT-SPE	Aux	88.3-	105.2	watermelon, mung bean sprouts	[52]
MSN@MIL-101(Fe)	dSPE	ABA, Aux	76.1-	113.0	mung bean sprouts	[53]
MOF-199/CNTs	SPME	ETH	86.8-	105.0	durian husk, wampee, blueberry, grape	[55]
CuTPA MOF	SPME	ETH	-		banana, avocado	[56]

-: not mentioned.

### 2.3 共价有机框架材料

共价有机框架(covalent organic frameworks, COFs)主要由轻质元素(C、H、O、N、B等)通过共价键连接而成,于2005年提出,成为继MOFs之后另一类重要的三维有序材料^[58]^。目前,COFs合成方法包括水热法、室温合成法、微波辅助法等。该类材料在PHs吸附萃取中的研究还相当稀少,尚有很大的应用前景。

根据共价键官能团不同,COFs材料细分为含硼类、三嗪类、亚胺类。其中,亚胺类COFs以醛胺脱水缩合发生席夫碱反应,合成条件简单、应用较为广泛^[59]^。2020年,Li等^[60]^以Fe_3_O_4_纳米颗粒为磁芯,1,3,5-三甲酰基间苯三酚(Tp)和2,6-二氨基蒽醌(DA)发生席夫碱缩合反应。合成Fe_3_O_4_@COF(TpDA)材料的壳层厚度约75 nm,比表面积高达180.2 m^2^/g。结合MSPE-HPLC-DAD方法,首次用于果蔬样品中7种Aux萃取,检出限介于4.68~7.51 ng/mL。

### 2.4 分子印迹聚合物

作为一类高选择性材料,分子印迹聚合物(molecularly imprinted polymer, MIP)在样品前处理领域应用广泛。中山大学李攻科课题组较早将MIP应用于Aux^[61,62]^、BRs^[63,64]^、GAs^[65]^等几类激素的特异性分析。Campanella等^[66]^在没有模板分子的情况下,开发了一种以4-乙烯基吡啶(4-VP)为功能单体、三羟甲基丙烷三甲基丙烯酸酯(TRIM)为交联剂的新型聚合物,能实现IAA特异性萃取。2018年,Li等^[67]^合成了纤维素磁性分子印迹聚合物微球,选择*β*-CD和4-VP两种功能单体来增强识别IAA的能力。类似地,Wang等^[68]^选择甲基丙烯酸(MAA)和*β*-CD功能单体,合成了聚合物*β*-CD/MAA-MIPs,对Aux的选择性吸附效果优于常规*β*-CD-MIPs及MAA-MIPs等材料,再次印证了双功能单体能提供更多吸附位点,有利于增强特异性识别能力。

在分子印迹树脂应用上,凹凸棒石/亲水性分子印迹整体树脂(AT/HMIMR)材料具有比表面积大、亲水性能优的特点,其中,凹凸棒石具有纳米多孔结构和一定的阳离子交换能力,作为PT-SPE吸附剂能实现植物样品中Aux选择性萃取^[69,70]^。Aihebaier等^[71]^提出微萃取棒分子印迹方法(MI-μ-SPE),通过在HF中填充MIP颗粒形成微萃取棒,借助HF的孔洞效应和保护作用,可以选择性地优先吸附IBA。结合HPLC方法对绿豆芽中IBA检出限为7.5 ng/g。

以KT为模板分子,Han等^[72]^提出离子液体杂化分子印迹材料-过滤头固相萃取法(IL-HIM-FSPE)测定豆芽中2种CTKs。Wang等^[73]^使用腺嘌呤为模板分子,开发了3-氨基苯酚-六亚甲基四胺(MIAPH)分子印迹树脂,用于选择性识别KT和BA,萃取效果优于HLB和C_18_等商品化吸附剂。本课题组将分子印迹和固相微萃取相结合,开发分子印记固相微萃取涂层(molecularly imprinted solid phase microextraction, MISPME)^[74,75,76,77]^,分别实现了CTKs和Aux两类激素的选择性萃取。结合HPLC-DAD检测法,对IAA和IPA的富集因子分别为158和146,对2种CTKs的检出限为0.3 ng/mL。自制MISPME涂层具有分子印迹识别、静电引力、*π-π*相互作用等多重吸附机理。

### 2.5 超分子化合物

超分子(supramolecular)化合物凭借空间匹配效应和主-客体分子识别能力,在样品前处理应用中一般作为吸附材料的改性剂。环糊精、冠醚、杯芳烃和葫芦脲等各类超分子化合物均能辅助提高目标物的萃取选择性^[78]^。基于*β*-CD“外亲水、内疏水”的分子结构,PHs很容易被吸附到立体疏水空腔中,经*β*-CD改性的CNTs、GO、MIP等复合材料萃取选择性都明显增强^[12,21-24,42,67,68]^。

杯芳烃被认为是第三代超分子化合物的典型代表。三嗪改性硅胶杯芳烃(NCS)是一种混合模式吸附剂,吸附机理包括空腔尺寸、疏水性、氢键、*π-π*作用等。NCS-SPE-HPLC方法已成功用于测定辣椒和小麦样品不同生长部位的IAA和IBA,选择性吸附行为和理论计算的结果相符合^[79]^。

作为第四代超分子化合物,葫芦脲(CB_[_*_n_*_]_, *n*=5~8,10,14等)呈桶形结构,拥有亲水性端口和疏水性空腔。该类化合物端口的羰基带负电,容易与带正电的有机物发生分子间相互作用。CTKs类激素含有腺嘌呤和苯基脲母环结构,呈现一定的正电性,适合被CB_[_*_n_*_]_选择性萃取。Zhang等^[80]^首先在无水条件下合成CB_[8]_材料,氮气保护下缓慢滴加3-异氰基酰氨基丙基三乙氧基硅烷,完成CB_[8]_功能化修饰形成过羟基葫芦脲(PCB_[8]_),再加入Fe_3_O_4_@SiO_2_微球,最终制备磁性的过羟基葫芦脲(MPC)材料,对CTKs的吸附量为10.15~18.28 nmol,富集因子大于200。该材料的吸附驱动力在于PCB_[8]_与分析物之间的主客体以及氢键相互作用。

## 3 总结和展望

新型固相吸附剂的研究是样品前处理研究领域的一项重要内容。综上所述,PHs种类丰富、结构多样,含有羟基、氨基、羧基、苯环等作用位点,并带有一定的极性和酸碱性。碳基材料、有机骨架化合物、分子印迹聚合物等材料都具有物化性能优异、比表面积大、易于结构改性等特点,适合应用于PHs的吸附萃取。它们之间的作用机理主要涉及疏水、氢键、*π-π*堆积、主客体识别等多重作用力。

然而,G材料的团聚问题导致吸附性能下降,碳氮材料制备过程存在缩聚不完全现象。部分MOFs(IRMOF系列)在空气或溶剂(水相)中不稳定,易导致结构坍塌或改变。COFs材料质量很轻,通过简单离心法较难彻底分离,限制了其在dSPE方法中应用。MIP技术中,模板分子流失、吸附过程较慢、萃取容量较低等问题亟待解决。超分子化合物较少能单独作为SPE有关方法的吸附材料。尽管这些不足使得上述材料作为SPE有关方法的吸附剂应用上受到一定限制,然而,通过进一步功能化修饰或者制备性能更为优异的复合材料是一个重要的改进途径。同时,如何实现上述材料的低成本生产和大规模应用,也是未来重要的一个发展方向。不可否认,上述材料在PHs分析萃取中仍将发挥越来越大的作用。

此外,PHs原位、超高灵敏度分析和时空分布研究逐渐热门。这要归功于分析方法(例如色谱-串联质谱技术配合各种衍生化增敏方法)和样品前处理技术(开发了性能更好的新方法和新材料)的同步发展,使得毫克级、亚毫克级鲜样甚至于单个叶片、花蕊、种子都能成为实际植物样品,PHs分析将朝着更小样品量、更高灵敏度、更高通量等方向发展。
